# Nationwide Analysis of Glaucoma Surgeries in Fiscal Years of 2014 and 2020 in Japan

**DOI:** 10.3390/jpm13071047

**Published:** 2023-06-26

**Authors:** Masaki Tanito

**Affiliations:** Department of Ophthalmology, Shimane University Faculty of Medicine, Izumo 693-8501, Shimane, Japan; mtanito@med.shimane-u.ac.jp; Tel.: +81-853-20-2284

**Keywords:** nationwide, NDB Open Data, glaucoma surgery, minimally invasive glaucoma surgery, selective laser trabeculoplasty, micropulse cyclophotocoagulation

## Abstract

Nationwide trends in glaucoma surgical procedures were assessed by using the NDB Open Data 2014 and 2020. In Japan, 33,340 non-laser, 54,569 laser, and 88,019 total glaucoma surgeries were performed in 2014. In 2020, 60,108 non-laser, 60,547 laser, and 120,655 total glaucoma surgeries were performed. The rates from 2014 to 2020 were 180%, 111%, and 137%, respectively. In each procedure, angle surgery (326%), tube shunt surgery (383%), ciliary coagulation (489%), and gonio-laser (225%) were remarkably increased, while iridectomy (75%) and iris laser (77%) decreased during the same period. An increase in laser surgery was seen in young age groups, namely, 55–59 years old and younger, while non-laser surgery was increased in old age groups, namely, 45–49 years old and older. In 2020, 47.6 non-laser, 48.0 laser, and 95.6 total glaucoma surgeries were performed per 100,000 persons. None of the vital statistics, including prefectural population, mean age, and rate of ≥65-year-old people, were significantly associated with the number of glaucoma surgeries. Glaucoma practice patterns changed each time a new device or procedure was introduced. The results of the current study reflected the use of new procedures, such as minimally invasive glaucoma surgery, tube shunt, selective laser trabeculoplasty, and micropulse cyclophotocoagulation.

## 1. Introduction

Glaucoma is one of the major causes of severe visual loss and blindness around the world [1]. The worldwide estimated prevalence of glaucoma is 3.54% in the population aged 40 to 80 years [2]. Among the global population with moderate or severe visual impairment (237.1 million), 3.2 million were expected to be suffering from glaucoma [3]. Intraocular pressure (IOP) above the physiologic levels is the main risk factor for developing glaucoma, and a reduction in IOP via medication, laser application, or incisional surgery may slow disease progression.

Because of the increase in the number of glaucoma patients due to an increase in the aged population [4], and because of the advent of new techniques, devices, and equipment that provides alternative treatments other than medication [5,6], the clinical indication of laser and other surgery should have changed dramatically in the past decade [7,8]. Accordingly, to understand the current status of glaucoma surgery, an updated analysis using the appropriate database was required.

The National Database of Health Insurance Claims and Specific Health Checkups of Japan (NDB), provided by the Ministry of Health and Welfare, contains all the insurance claims of the Japanese national healthcare system; representative data, including nationwide surgical claims, are public as NDB Open Data (https://www.mhlw.go.jp/stf/seisakunitsuite/bunya/0000177182.html; accessed on 1 June 2023). In this manuscript, by using the oldest (fiscal year 2014) and newest (fiscal year 2020) databases of NDB Open Data, the nationwide trends of glaucoma treatment other than medication were analyzed.

## 2. Subjects and Methods

This study adhered to the tenets of the Declaration of Helsinki. Based on the Ethical Guidelines for Medical and Health Research Involving Human Subjects in Japan, given that the study included open-source data only, ethical approval was not required. The first set of NDB Open Data, which contained data from April 2014 to March 2015, and the seventh set of NDB Open Data, which contained data from April 2020 to March 2021, were used. The database reported the number of glaucoma surgeries claimed by Japanese governmental health insurance in the respective fiscal years. The number of surgeries was aggregated by code K268-1 for iridectomy, K268-2 (including trabeculotomy, goniotomy, and goniosynechialysis (GSL)) and K268-6 (combined cataract and iStent implantation) for angle surgery, K268-3 (trabeculectomy) and K268-4 (ExPRESS shunt) for filtration surgery, K268-5 (including Ahmed glaucoma valve and Baerveldt glaucoma implant) for tube shunt surgery, K271 (including transscleral cyclophotocoagulation (CPC), and micropulse cyclophotocoagulation (MCP)) and K272 (cyclocryotherapy) for ciliary coagulation, K270 (including laser iridotomy (LI) and laser gonioplasty) for iris laser, and K273 (including laser trabeculoplasty (LTP) and selective laser trabeculoplasty (SLT)) for gonio-laser (Table 1, aggregation 1). The surgeries were also aggregated by codes K268-1, K268-2, K268-3, K268-4, K268-5, K268-6, and K272 for non-laser surgery (i.e., incisional surgeries and cyclocryotherapy), and K270, K271, and K273 for laser surgery (Table 1, aggregation 2). In the national insurance system of Japan, only one glaucoma procedure can be claimed, even in a case where multiple glaucoma procedures were performed at once (e.g., combination of trabeculotomy and trabeculectomy); it was up to the surgeon/hospital to decide which procedure to claim. In the NDB Open Data, the numbers of each surgical procedure were provided in 5-year steps for the age of patients or each of the 47 prefectures in Japan; the number of surgeries fewer than 10 in each age class or prefecture was omitted; therefore, the total number of surgeries were not completely the same between age- and prefecture-based aggregation. The percent change in the numbers of glaucoma surgeries were obtained via a comparison between 2014 and 2020.

To calculate the number of glaucoma surgeries per 100,000 capita, the populations of the 47 prefectures from the national census in 2020 provided by the Statistics Bureau of Japan (https://www.stat.go.jp/english/index.html; accessed on 1 June 2023) were used. Possible correlations between the per 100,000 capita glaucoma surgeries and prefectural population statistical parameters (i.e., prefectural population, mean age, and rate of population older than 65 years or older) were assessed with Spearman’s correlation coefficient using the 2020 database.

All the statistical analyses were performed using JPM Pro statistical software version 15.2 (SAS Institute, Inc., Cary, NC, USA). The established datasets are available as Supplementary files (Tables S1 and S2).

## 3. Results

The number of aggregated glaucoma surgeries is summarized in Table 2. In Japan, 33,340 non-laser, 54,569 laser, and 88,019 total glaucoma surgeries were performed in 2014. In 2020, 60,108 non-laser, 60,547 laser, and 120,655 total glaucoma surgeries were performed. The changes from 2014 to 2020 (no change = 100%) were 180%, 111%, and 137%. In each procedure, angle surgery (326%), tube shunt surgery (383%), ciliary coagulation (489%), and gonio-laser (225%) were remarkably increased, while iridectomy (75%) and iris laser (77%) decreased during the same period.

**Figure 1:**
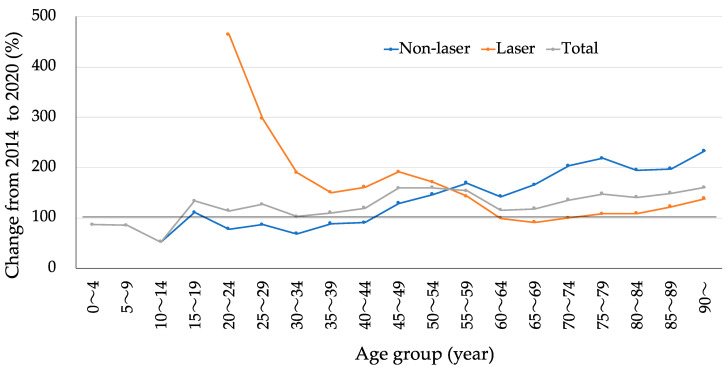
Change in the number of glaucoma surgeries from 2014 to 2020 in each age group (no change = 100%).

The changes in surgical numbers from 2014 to 2020 in each age group are shown in Figure 1 (no change = 100%). An increase in laser surgery was seen in young age groups, namely, 55–59 years old and younger (orange line), while non-laser surgery was increased in old age groups, namely, 45–49 years old and older (blue line). As a result, the rate of total glaucoma surgery was more than 100% in all age groups, except for 10–14 years old and younger age groups (gray line).

In Japan, 47.6 non-laser, 48.0 laser, and 95.6 total glaucoma surgeries were performed per 100,000 persons in 2020 (Table 2). To assess the role of age on the number of glaucoma surgeries, the data on the number of surgeries and vital statistics from 47 prefectures in Japan were used. Table 3 summarized the association analysis between the per capita number of glaucoma surgeries and prefectural vital statistics. Somewhat unexpectedly, none of the vital statistics, including the prefectural population, mean age, and proportion of ≥65-year-old people, were significantly associated with the number of glaucoma surgeries, although the association between the number of per capita laser surgeries and the prefectural population was borderline (ρ = 0.26, *p* = 0.08).

**Figure 2:**
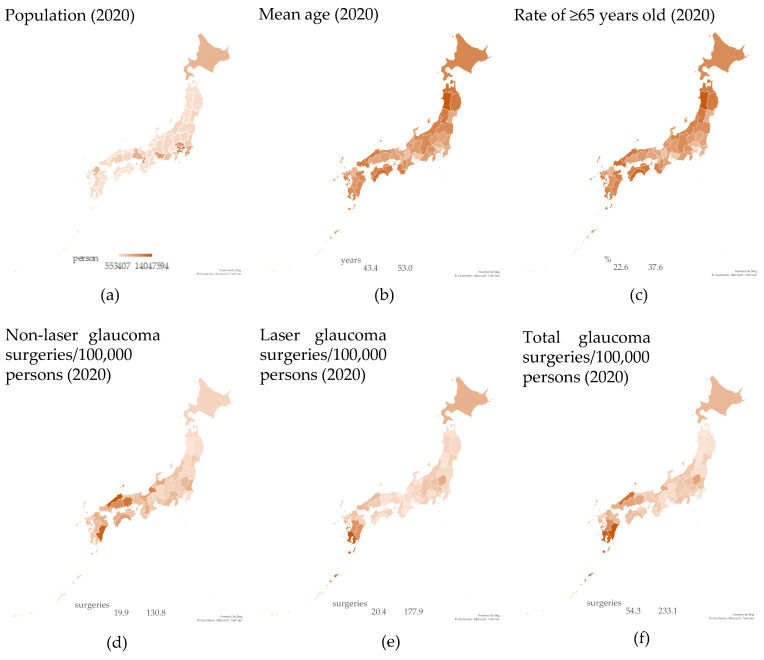
Geographical distribution of the prefectural population (**a**), mean age (**b**), and rate of the older population (**c**), as well as the per 100,000 capita non-laser (**d**), laser (**e**), and total (**f**) glaucoma surgeries in 47 Japanese prefectures in 2020.

The geographical distributions of the parameters analyzed are shown in Figure 2. Regarding the vital statistical parameters, opposite distributions for population (a) and mean age (b)/older age rate (c) (i.e., the aging rate was higher where the population was lower, and vice versa) were clearly seen. On the other hand, the distribution of the numbers of non-laser (d), laser (e), and total (f) surgeries seemed to be distributed independently of the vital statistical parameters (a–c); this coincided well with the analysis shown in Table 3.

## 4. Discussion

In this study, nationwide trends in glaucoma surgical procedures were assessed by using the NDB Open Data; as a result, it was revealed that the numbers of non-laser, laser, and total surgeries increased by 180%, 111%, and 137%, respectively, from 2014 to 2020 in Japan. Previously, using the Diagnostic Procedure Combination database, Japanese trends in glaucoma surgery between 2011 and 2019 were reported [9]. A previous report found 14,163 glaucoma-related procedures in 2014 [9]; the number of procedures aggregated in this study was 6.2 times more in that year, thus the current study might cover a wider range of the real-world situation. In France, by analyzing the national database of the French Diagnosis-Related Group system between 1997 and 2000, new glaucoma drugs, primarily latanoprost and brimonidine, improved IOP control, and delayed surgery reduced the glaucoma procedure rate in patients receiving glaucoma-related medical treatment by 47% [10]. In the Netherlands, by analyzing the Dutch Healthcare Registration from 1995 to 2003, the number of glaucoma surgeries started to decrease, resulting in a 45% decrease in the year 2000 compared with 1995, while the total number of prescriptions rose by 20% in the year 1999 compared with 1998 and then stabilized [11]. Accordingly, the decrease in glaucoma surgery is most likely explained by the introduction of new medications during the relevant period. In Australia, by analyzing the Health Insurance Commission (i.e., Medicare) database between 1994 and 2003, the rates of LTP and trabeculectomy surgeries fell by 60% and 58%, respectively; the introduction of multiple new medications resulted in a decline in the number of glaucoma surgeries and LTPs performed [12]. Thus, the trends may be different between the recent decade and 2–3 decades ago, suggesting the importance of continuous updates to keep abreast of recent trends.

In this study, the increase in non-laser surgery was remarkable; this was due to the increase in angle surgery (326%) and tube shunt surgery (383%). In the United States, by analyzing Medicare paid claims data between 1994 and 2012, despite a 9% increase in beneficiaries, the total number of glaucoma procedures and the number of glaucoma procedures other than laser procedures decreased by 16% and 31%, respectively [13]. More recently, in the United States, by using the American Academy of Ophthalmology Intelligent Research in Sight Registry, a substantial increase in the annual minimally invasive glaucoma surgery (MIGS) procedures occurred over the study period (from 7586 in 2013 to 39,677 in 2018), as well as a smaller decrease in standard glaucoma procedures (from 16,215 to 13,701 in 2018) [14]. In Germany, by analyzing the quality reports of hospitals, it was found that the number of glaucoma procedures performed increased by 75% from 27,811 in 2006 to 48,794 in 2018 [15]. The numbers of trabeculectomies, goniotomies, and ab externo trabeculotomies decreased between 2006 and 2018, while the use of MIGS has increased since 2012, suggesting a trend toward the modern surgical options and especially MIGS during recent years [15]. In the United States, among Medicare beneficiaries from 2008 to 2016, a large shift in practice from traditional incisional glaucoma surgeries to MIGS procedures was observed [16]. During the relevant period, glaucoma specialists continued to perform mostly traditional incisional glaucoma surgeries, while many MIGS procedures were performed by other-than-glaucoma specialists [16]. Another study in Japan reported that the total number of glaucoma-related procedures increased throughout the years 2011 to 2019; before 2017, filtering surgery was the most common procedure, whereas trabeculotomy was the most common after 2018 [9]. An increase in the number of non-laser procedures seen in middle age and older age groups (Figure 1) can be explained by the use of MIGS procedure in these age groups since MIGS is frequently performed in combination with cataract surgery [6,17]. Accordingly, the results of this study coincided well with the global trends [18].

In this study, the increase in laser surgery was due to the increases in ciliary coagulation (489%) and gonio-laser (225%). In 1998, the first successful protocol of SLT was established for lowering the IOP in the treatment of open-angle glaucoma [19]. Before the clinical use of SLT, by analyzing Ontario Health Insurance Plan billing service claims between 1992 and 2012 in Canada, an overall increase in the rate of gonio-laser, no change in the rate of trabeculectomies, and a significant increase in tube shunt surgery were found [20]. In a later study conducted in Canada, there was a substantial reduction in the number of gonio-laser procedures between 1997 and 2001; however, the gonio-laser rates increased between 2002 and 2004, coinciding with the introduction of SLT [21]. Recent studies demonstrated the safety and efficacy of SLT as an early treatment option for glaucoma [22,23]. The European Glaucoma Society has recommended SLT as the first-line or adjunctive treatment in open-angle glaucoma and ocular hypertension, reiterating its clinical significance [24,25]. In addition to the CPC using a continuous wave, the device using a micropulse was approved in 2017 in Japan. It now appears that the safety profile of the micropulse procedure is superior to that of the continuous wave procedure [26]. Thus, the introduction of MCP likely explains the dramatic increase in ciliary coagulation during the period of this study. In Australia, by analyzing Medicare between 2003 and 2017, there were significant increases in laser procedure rates, including gonio-laser (4.61-fold), LI (2.55-fold), and ciliary coagulation (2.33-fold) [27]. The increase in laser procedures in younger age groups in this study (Figure 1) can be explained by the early indication of recently introduced laser procedures, such as SLT and MCP, in these age groups.

In this study, iridectomy (75%) and iris laser (77%) procedures decreased during the study period. In Korea, by analyzing the Korean Health Insurance Review and Assessment service database between 2007 and 2012 regarding the treatment of primary angle closure (PAC), LI manifested a slow decline (17% decrease), and cataract surgery showed a sharp rise (49% increase) [28]. In Japan, for the treatment of primary angle closure glaucoma (PACG), the amount of antiglaucoma medications dispensed decreased and the frequency of cataract surgery increased, while the frequency of laser LI procedures decreased between 2010 and 2020 [29]. This agreed well with the switch of treatment recommendation for PAC/PACG from LI to lens removal [30]. In Germany, iridotomies and iridectomies increased by 120% and 248%, respectively, over the period between 2006 and 2018 [15]. In Scotland, there was a significant reduction in the rate of acute PACs between 1998 and 2012, along with a rising rate of LI and cataract surgeries; the trend of decreasing acute PACs may have been due to the increasing rate of cataract surgery in the same period [31]. Thus, other than the difference in study periods, epidemiological factors, such as a difference in the aging of the population and race, might explain the different trends of iridotomy between this Japanese study and previous reports from European countries. In this aggregation, GSL that was frequently combined with cataract surgery for treatment of PAC/PACG was counted as the angle surgery; accordingly, an increase in GSL may also have contributed to the overall increase in angle surgery between 2014 and 2020 in this study. On the other hand, when the cataract surgery alone was performed for the treatment of PAC/PACG, the procedure did not appear in the dataset as a glaucoma procedure; therefore, the treatment frequencies for PAC/PACG should be underestimated in this study.

The per capita number of glaucoma surgery and correlation assessment between the prefectures/provinces and the number of glaucoma surgeries reported in this study are relatively unique in the literature. In Portugal, by analyzing the Portuguese national hospitalization database, the numbers of non-laser glaucoma surgeries per 100,000 population were 11.2, 11.0, 15.2, and 20.4 in the years 2000, 2005, 2010, and 2015, respectively [32]. In Scotland, England, and Wales, the overall trabeculectomy rates per 100,000 persons remained stable from 9.06 in 2003 to 10.76 in 2012 [33]. In Australia, the numbers of trabeculectomies per 100,000 persons were 15.1 in the year 2001 and 13.2 in 2018, while for tube shunt surgery, this was 0.6 in the year 2001 and 13.3 in 2018 [34]. None of the prefectural vital statistics were significantly associated with the number of glaucoma surgeries. Accordingly, other than the aging of the population, factors such as the distributions of hospitals and glaucoma specialists can be strong determinants of the number of glaucoma procedures. In general, health systems that remunerate by procedure (more than by outpatient attendance) tend to have higher procedure levels per capita than those that remunerate in a more balanced way between clinic visits and procedures. The Japanese health system remunerates for each procedure, and thus, other than based on the clinical evidence, decisions according to remuneration opportunity might drive the recent increase in glaucoma procedures to some degree. Recent reports from Japan suggested that combined MIGS and cataract surgery could be chosen in glaucoma patients who had not been treated with medication [35]. The study site of that report was the tertiary care hospital of the prefecture that had the lowest population, and the patients were referred from far and wide. Accordingly, the difference in the ease of access to specialized care and/or the physicians’ practice pattern might be affecting the patients’ preference for therapy.

The analyses presented in this study were solely based on the NDB Open Data, and that data is a simple aggregation of the claims made to the national insurance system of Japan. As indicated previously, only a single procedure can be claimed, even in a case where multiple glaucoma procedures were performed at once; thus, the counts presented in this study can be considered to be a minimum number of glaucoma surgeries performed in the relevant period. As also indicated previously, the database analyzed excluded the procedures with fewer than 10 of each procedure in a 5-year age step for each sex or each prefecture. Thus, errors may be large in categories with small numbers of cases, such as young age groups. The data provided by the NDB open data are not linked to each other, and no clinical background was provided in this dataset; therefore, it is not possible to perform further analyses, such as the subgroup aggregation based on whether a claim was for an initial or secondary surgery and with or without simultaneous cataract surgery. In the same context, subgroup analyses based on the glaucoma types stratified by primary or secondary and open or closed angle also cannot be performed using this database. Glaucoma practice patterns change each time a new device or procedure is introduced [36]. The results of the current study reflect the use of new procedures, such as MIGS, tube shunt, SLT, and MCP.

## 5. Conclusions

Trends in glaucoma surgeries in Japan were analyzed using the largest database provided by the Japanese government. The results provide the real-world trends in glaucoma surgery in a country that has the highest aging rate in the world (https://www.stat.go.jp/data/topics/topi1261.html, accessed on 1 June 2023).

## Figures and Tables

**Table 1:** Classification of procedures.

Code	Representative Procedures	Aggregation 1	Aggregation 2
K268-1	Iridectomy	Iridectomy	Non-laser
K268-2	Trabeculotomy ab externo, Trabectome, KDB, Tanito microhook, GATT, GSL	Angle surgery	
K268-6	iStent		
K268-3	Trabeculectomy, NPT	Filtration surgery	
K268-4	ExPRESS shunt		
K268-5	Ahmed, Bearveldt	Tube shunt	
K272	Cyclocryotherapy	Ciliary coagulation	
K271	Cyclophotocoagulation, micropulse cyclophotocoagulation		Laser
K270	LI, LGP	Iris laser	
K273	LTP, SLT	Gonio-laser	

KDB, Kahook dual blade; GATT, gonioscopy-assisted transluminal trabeculotomy; GSL, goniosynechialysis; NPT, non-penetrating trabeculectomy; LI, laser iridotomy; LGP, laser gonioplasty; LTP, laser trabeculoplasty; SLT, selective laser trabeculoplasty.

**Table 2:** Number of glaucoma surgeries in 2014 and 2020.

Procedure	2014	2020	Change (%)
Aggregation 1			
Iridectomy	1608	1209	75
Angle surgery	10,957	35,759	326
Filtration surgery	19,844	19,909	100
Tube shunt	817	3129	383
Ciliary coagulation	631	3085	489
Iris laser	43,518	33,661	77
Gonio-laser	10,644	23,903	225
Aggregation 2			
Non-laser	33,340	60,108	180
Laser	54,679	60,547	111
Total	88,019	120,655	137
Per 100,000 capita			
Non-laser	-	47.6	-
Laser	-	48.0	-
Total	-	95.6	-

**Table 3:** Correlations between the per 100,000 capita glaucoma surgeries and population statistical parameters among the 47 Japanese prefectures in 2020.

Parameters	Prefectural Population (Person)		Prefectural Mean Age (Years)		Prefectural Rate of ≥65-Year-Olds (%)	
Non-laser/100,000 persons	ρ = −0.17	*p* = 0.24	ρ = 0.01	*p* = 0.95	ρ = 0.08	*p* = 0.58
Laser/100,000 persons	0.26	0.08	−0.02	0.92	0.01	0.93
Total/100,000 persons	0.06	0.67	0.02	0.9	0.08	0.58

The ρ and *p* values were calculated with Spearman’s correlation coefficient using 2020 database.

## Data Availability

All the relevant data used in this study are included in this manuscript or available as Supplementary Tables S1 and S2.

## Supplementary Materials

The following supporting information can be downloaded at https://www.mdpi.com/article/10.3390/jpm13071047/s1; (accessed om 1 June 2023). Table S1. Full dataset stratified by age group; Table S2. Full dataset stratified by prefecture.
