# Supplementary material for: Nationwide Analysis of Glaucoma Surgeries in Fiscal Years of 2014 and 2020 in Japan
Source: J Pers Med. 2023 Jun 26;13(7):1047. doi: 10.3390/jpm13071047 (PMC10381819; doi:10.3390/jpm13071047)
Supplement: Supplementary file 1 [file jpm-13-01047-s001.zip › Table S1.pdf]

Table S1. Data by age group

Age group	FY2014 Iridectomy	Angle-surgery	Filtration	Tube shunt	Ciliary coagulation	Iris laser	Gonio laser	Non-laser	Laser	Total	FY2020 Iridectomy	Angle-surgery	Filtration	Tube shunt	Ciliary coagulation	Iris laser	Gonio laser	Non-laser	Laser	Total	Change Iridectomy	Angle-surgery	Filtration	Tube shunt	Ciliary coagulation	Iris laser	Gonio laser	Non-laser	Laser	Total
Total	1608	10957	19844	817	631	43518	10644	33340	54679	88019	1209	35759	19909	3129	3085	33661	23903	60108	60547	120655	75.2	326.4	100.3	383.0	488.9	77.3	224.6	180.3	110.7	137.1
0～4	0	137	0	0	0	0	0	137	0	137	0	107	0	11	0	0	0	118	0	118		78.1						86.1		86.1
5～9	0	34	0	0	0	0	0	34	0	34	0	18	0	11	0	0	0	29	0	29		52.9						85.3		85.3
10～14	0	44	0	0	0	0	0	44	0	44	0	23	0	0	0	0	0	23	0	23		52.3						52.3		52.3
15～19	0	57	28	10	0	0	0	95	0	95	0	63	42	0	0	0	22	105	22	127		110.5	150.0	0.0				110.5		133.7
20～24	0	89	45	0	0	0	14	134	14	148	0	62	42	0	0	11	54	104	65	169		69.7	93.3				385.7	77.6	464.3	114.2
25～29	0	64	67	10	0	19	14	141	33	174	0	71	51	0	10	23	65	122	98	220		110.9	76.1	0.0		121.1	464.3	86.5	297.0	126.4
30～34	0	101	143	13	0	53	47	257	100	357	0	82	69	24	29	40	121	175	190	365		81.2	48.3	184.6		75.5	257.4	68.1	190.0	102.2
35～39	0	132	293	31	15	127	102	456	244	700	0	179	173	50	21	104	241	402	366	768		135.6	59.0	161.3	140.0	81.9	236.3	88.2	150.0	109.7
40～44	15	205	453	41	16	282	189	714	487	1201	0	278	276	91	48	270	466	645	784	1429	0.0	135.6	60.9	222.0	300.0	95.7	246.6	90.3	161.0	119.0
45～49	31	238	644	48	25	594	274	961	893	1854	17	489	599	133	124	724	860	1238	1708	2946	54.8	205.5	93.0	277.1	496.0	121.9	313.9	128.8	191.3	158.9
50～54	46	297	980	46	49	1130	423	1382	1589	2971	29	902	943	151	124	1339	1259	2025	2722	4747	63.0	303.7	96.2	328.3	253.1	118.5	297.6	146.5	171.3	159.8
55～59	80	460	1314	58	44	2063	587	1912	2694	4606	73	1522	1418	215	196	2111	1538	3228	3845	7073	91.3	330.9	107.9	370.7	445.5	102.3	262.0	168.8	142.7	153.6
60～64	148	857	2152	86	49	4412	818	3243	5279	8522	99	2418	1849	240	209	3190	1832	4606	5231	9837	66.9	282.1	85.9	279.1	426.5	72.3	224.0	142.0	99.1	115.4
65～69	225	1353	2899	132	58	7041	1177	4609	8276	12885	131	4465	2705	346	303	4733	2470	7647	7506	15153	58.2	330.0	93.3	262.1	522.4	67.2	209.9	165.9	90.7	117.6
70～74	287	2160	3536	102	59	9649	1886	6085	11594	17679	228	7762	3847	546	436	7282	3820	12383	11538	23921	79.4	359.4	108.8	535.3	739.0	75.5	202.5	203.5	99.5	135.3
75～79	275	2084	3284	88	64	8419	1770	5731	10253	15984	194	8129	3663	534	461	6274	4304	12520	11039	23559	70.5	390.1	111.5	606.8	720.3	74.5	243.2	218.5	107.7	147.4
80～84	234	1693	2531	79	73	5839	1774	4549	7674	12223	170	5741	2570	378	456	4282	3582	8869	8310	17179	72.6	339.1	101.5	478.5	624.7	73.3	201.9	195.0	108.3	140.5
85～89	144	785	1221	31	53	2859	1129	2191	4031	6222	127	2720	1247	227	301	2367	2255	4321	4923	9244	88.2	346.5	102.1	732.3	567.9	82.8	199.7	197.2	122.1	148.6
90～	71	167	195	0	10	1008	410	433	1428	1861	24	636	312	38	135	867	967	1010	1969	2979	33.8	380.8	160.0		1350.0	86.0	235.9	233.3	137.9	160.1
