# Supplementary material for: Nationwide Analysis of Glaucoma Surgeries in Fiscal Years of 2014 and 2020 in Japan
Source: J Pers Med. 2023 Jun 26;13(7):1047. doi: 10.3390/jpm13071047 (PMC10381819; doi:10.3390/jpm13071047)
Supplement: Supplementary file 1 [file jpm-13-01047-s001.zip › Table S2.pdf]

Table S2. 2020 data by prefecture

Prefecture	Population	mean age	≥65 years	Non-laser	Laser	Total
Hokkaido	5224614	49.8	32.2	33.4	78.5	111.9
Aomori	1237984	50.9	33.9	28.4	26.2	54.6
Iwate	1210534	50.7	33.8	25.7	34.2	59.9
Miyagi	2301996	47.5	28.3	38.8	43.6	82.5
Akita	959502	53.0	37.6	19.9	38.0	57.9
Yamagata	1068027	50.6	34.0	30.2	24.1	54.3
Fukushima	1833152	49.6	31.8	30.4	33.2	63.6
Ibaraki	2867009	48.2	29.9	60.0	35.4	95.5
Tochigi	1933146	48.0	29.2	55.0	82.6	137.7
Gunma	1939110	48.5	30.4	38.5	58.8	97.3
Saitama	7344765	46.9	27.1	38.9	61.3	100.2
Chiba	6284480	47.1	27.6	24.9	31.9	56.8
Tokyo	14047594	45.3	22.8	36.9	53.7	90.6
Kanagawa	9237337	46.5	25.6	40.0	38.6	78.6
Niigata	2201272	50.0	32.9	45.2	47.1	92.2
Toyama	1034814	49.8	32.8	67.8	23.3	91.1
Ishikawa	1132526	48.0	30.0	46.4	40.9	87.3
Fukui	766863	48.5	30.8	97.1	27.1	124.3
Yamanashi	809974	49.1	31.1	56.1	27.8	83.8
Nagano	2048011	49.6	32.2	35.5	55.5	91.0
Gifu	1978742	48.4	30.6	28.7	37.0	65.6
Shizuoka	3633202	48.4	30.2	54.1	43.0	97.1
Aichi	7542415	45.7	25.4	44.2	30.0	74.2
Mie	1770254	48.4	30.2	43.4	24.6	68.0
Shiga	1413610	45.9	26.4	35.3	29.7	65.0
Kyoto	2578087	47.8	29.4	66.9	22.8	89.8
Osaka	8837685	47.0	27.5	53.3	48.2	101.5
Hyogo	5465002	47.9	29.3	50.4	36.3	86.8
Nara	1324473	49.0	31.7	66.7	20.4	87.1
Wakayama	922584	50.2	33.4	41.1	32.8	73.9
Tottori	553407	49.3	32.5	43.4	32.7	76.1
Shimane	671126	50.2	34.4	130.8	69.0	199.8
Okayama	1888432	48.2	30.7	107.2	23.9	131.2
Hiroshima	2799702	47.7	29.6	86.9	50.1	137.0
Yamaguchi	1342059	50.5	34.8	55.6	69.9	125.5
Tokushima	719559	50.7	34.5	45.3	43.1	88.4
Kagawa	950244	49.1	31.9	50.0	23.7	73.7
Ehime	1334841	50.1	33.4	62.4	45.0	107.4
Kochi	691527	51.3	35.6	78.2	26.3	104.6
Fukuoka	5135214	46.7	28.1	60.1	57.6	117.7
Saga	811442	48.1	30.8	33.8	44.9	78.6
Nagasaki	1312317	49.7	33.1	49.5	54.6	104.1
Kumamoto	1738301	48.6	31.6	53.0	111.8	164.8
Oita	1123852	49.8	33.5	63.5	42.9	106.4
Miyazaki	1069576	49.3	32.7	122.8	110.3	233.1
Kagoshima	1588256	49.4	32.8	42.0	177.9	219.9
Okinawa	1467480	43.4	22.6	33.0	31.7	64.7
